# Editorial: Methods and protocols in nanotoxicology

**DOI:** 10.3389/ftox.2022.1093765

**Published:** 2022-12-15

**Authors:** Harald F. Krug, Katja Nau

**Affiliations:** ^1^ NanoCASE GmbH, Engelburg, Switzerland; ^2^ Karlsruhe Institute of Technology (KIT), Karlsruhe, Germany

**Keywords:** nanotoxicology, protocols, interference, genotoxicity, reproducibility

Although the first studies on the toxicology of nano-scale materials (colloids) were carried out nearly 100 years ago, the enormous increase in the number of studies on nanomaterials only began with the euphoria triggered by the targeted manipulation of matter at the atomic level. As a consequence, major concerns have been raised about the risks behind this technology (Hoet et al., 2004; Stern and McNeil, 2008). National or international initiatives or action plans have been established in many countries (cf. the National Nanotechnology Initiative [NNI], launched in 2000 in the United States, and the European Commission’s report *Nanosciences and Nanotechnologies: an action plan for Europe 2005–2009*, published in 2005). All of these initiatives contained funding programs focused on health and environmental impacts of nanomaterials. This circumstance led to a dramatic increase in the number of materials studied as well as publications on the biological safety of these materials (Figure 1). It quickly became obvious that nanomaterials pose a lot of problems when tested in biological assays. To be mentioned here are the interferences of the material with the test itself (Wörle-Knirsch et al., 2006; Kroll et al., 2012; Guadagnini et al., 2015). Furthermore, although the name is often identical (e.g., carbon nanotubes), the materials used are very different (e.g., single walled, multi-walled, short or long fibers, rigid and stiff or flexible and entangled), which makes an intensive characterization necessary in order to be able to classify the results correctly (Warheit, 2008; Crist et al., 2013). In addition, materials that have been on the market for a long time were hardly perceived as “nanomaterials” (e.g., TiO_2_, SiO_2_, carbon black), but these are now under discussion although registered as market products, such as TiO_2_.

**FIGURE 1:**
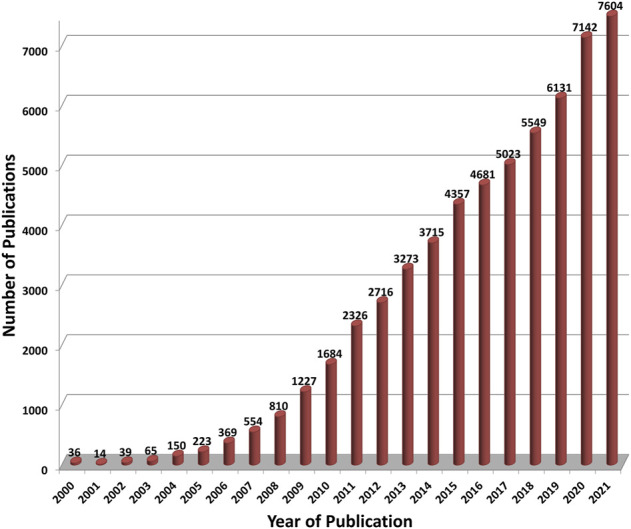
The number of publications on “Nanotoxicology” *per* year as found in the meta-database PubMed (https://pubmed.ncbi.nlm.nih.gov/). For each year from 2000 to 2021 (abscissa) publications have been searched within this database with the following search profile: “all fields” contain “nanotox*” or “fulleren* AND toxic*” or “nanotube AND toxic*” or “nanoparticle* AND toxic*” or “nanomat* AND toxic* or “nano* AND toxic*” or “graphene AND toxic” where the asterisk is a wild card.

In view of the enormous number of publications on nanotoxicology (> 60.000 since 2000, see Figure 1), the critical questions must be addressed: why is there still so much uncertainty in the statements on possible biological effects and why are results so inconsistent? Especially the reproducibility of results is in many cases very weak, although this is not restricted to nanotoxicology (Baker, 2016). Various scientists have criticized this situation (Hirsch et al., 2011; Krug, 2014; Petersen et al., 2014). Others tried to give answers and made suggestions for better reproducibility (Petersen et al., 2020), for enhancing the overall quality of studies (Fernández-Cruz et al., 2018) and “to generate inherently FAIR^1^ nanosafety data to support the efficient governance and regulation of nanomaterials” (Jeliazkova et al., 2021). In this context, there are still very important demands that have not yet been adequately met, despite the major funding programs and many national and international projects. This is because there is still a lack of harmonized protocols that are accepted in the scientific community. So far, some of the OECD test guidelines for the testing of chemicals have been adapted for nanomaterials, but the numerous protocols established and standardized in European projects are mostly not used in the in-depth study of nanomaterial toxicity in many laboratories. In fact, there are several European activities (e.g., the Malta Initiative^2^) and projects which contribute to the development or adaptation of OECD test guidelines for nanomaterials (e.g., NANOHARMONY^3^, Gov4Nano^4^, NANORIGO^4^, RiskGONE^4^). The German project DaNa has compiled a collection of standard operating procedures (SOPs) and laboratory protocols from different initiatives and published them online. In various subcategories, such as Biological Test Methods, Physico-Chemical Properties, Sample Preparation, one can download SOPs and laboratory protocols as pdf files. In addition, the DaNa team has set up a template with filling-in help for the creation of SOPs^5^. This pioneering activity is now complemented by EU projects and an SOP handbook is available at the website of the Horizon 2020 project PATROLS^6^.

For this reason, project activities are launched and journal special issues like this one are published to help improve the situation. The study of nanomaterials and their potential biological effects usually starts with *in vitro* cytotoxicity assays, which may be misleading because of interferences between the tested material and the assay components, as has been shown previously (Wörle-Knirsch et al., 2006). To overcome these problems, the Alamar Blue assay was further developed so that it can be reproducibly applied even in high-throughput experiments (Longhin et al.). A second example as an alternative for viability measurement is the colony-forming efficiency assay. This viability assay has been optimized for high-throughput experiments as well and is practically interference-free as no dyes are used. Moreover, the treatment time can be prolonged up to 10 days which can be regarded as a sub-chronic assay (Runden-Pran et al.). As a next step in the *in vitro* toxicity assessment the induction of oxidative stress is an important pathway of toxicity. Most often this endpoint is analyzed by using the fluorescence dye DCF, but this assay is like other fluorescence dye-dependent assay systems error-prone (Petersen et al., 2020). Alternatively, a better and more reliable analysis can be performed *via* the expression of anti-oxidative enzymes under the control of the nuclear erythroid 2-related factor 2 (NRF2) transcription factor. A world-wide consortium has developed a reporter gene assay for the measurement of NRF2 mediated gene expression and validated it *via* intra- and interlaboratory round robins (Martin et al.). Although the variability of the intra- and inter-laboratory results is relatively low, it becomes obvious that the higher the induction of expression, the higher is the variability between the labs.

The genotoxicity potential of TiO_2_ has recently been (re) evaluated but is still under discussion, and this clearly demonstrates the need for better and more reliable genotoxicity testing. Since this is the most important endpoint in the toxicological evaluation of a substance, the further development of existing assays and the establishment of new reliable tests is essential. Until now, the Comet assay has been criticized for being error-prone and providing biased results (Rajapakse et al., 2013; Ferraro et al., 2016). To avoid these weaknesses, an improved protocol was established that takes into account both cytotoxicity and uptake of nanoparticles by cells and establishes clear test acceptance criteria and consideration of historical controls (El Yamani et al.). The use of this approach will make the *in vitro* Comet assay much more reliable in the future. A similar study has set itself the task of adapting the existing protocol for the *in vivo* comet assay (OECD test guideline 489) so that the protocol can also be used for nanomaterials (Cardoso et al.). A comparable goal was set by another group. Here, the OECD test guideline 490 (thymidine kinase gene mutation test) was adapted for testing of nanomaterials. Also, with these changes to the existing protocol, care was taken to ensure that there are clear acceptance criteria and that the specific nanomaterial-related properties are considered (Chen et al.). It remains to be hoped that these adjustments to the existing OECD guidelines may be accepted by the scientific community and incorporated into the official protocols as soon as possible. The results of nanomaterial genotoxicity studies are often misleading as no discrimination between primary and secondary genotoxicity has been done. However, because many nanomaterials can induce oxidative stress or inflammatory processes that then indirectly lead to subsequent DNA damage, secondary DNA damage is often underestimated, and the overall genotoxicity of nanomaterials is overestimated. To better capture this shift in results, a co-culture system was established that discriminates well between primary and secondary genotoxicity (Vallabani and Karlsson). Using the example of nickel oxide nanoparticles with corresponding positive controls, it was shown that the cells used react significantly differently and human bronchial epithelial cells show exclusively secondary DNA damage. A further advantage of this protocol is the analysis of micronuclei by means of flow cytometry which reduces the possible bias. Taken together, with regard to genotoxicity, the articles in this special issue refer to various difficulties in the different steps of the individual methods when nanomaterials have to be investigated. As a kind of overview, another article in this series therefore addresses precisely these problems step by step and gives clear recommendations for avoiding them (Elespuru et al.). This article does not criticize the methodological errors of previous studies (what is wrong) but shows how the individual publication in this series addresses the critical points with positive advice to avoid these errors (what is right). The final article in this series covers a more basic aspect: establishing more realistic *in vitro* test systems. By using a microfluidic serum-free cell-on-a-chip system, it could be shown that dynamic conditions may reflect the tissue response in a more accurate way (Gupta et al.). In the future, such microfluidic systems are likely to encompass multiple cell types that can represent an entire organ, and may help to reduce animal testing, and increase the significance of *in vitro* approaches.

The methods and protocols presented in this special issue are intended to help improving transferability and reproducibility of results from different laboratories. Many potential sources of error have been identified and interferences of nanomaterials with assay systems have been demonstrated. The same applies to nanomaterials as for other chemicals: toxicological data are only useful and usable if they are confirmable by other laboratories.

## Footnotes

1 FAIR: findable, accessible, interoperable, reusable data.

2
https://www.nanosafetycluster.eu/international-cooperation/the-malta-initiative/.

3
https://nanoharmony.eu/.

4
https://www.nanosafetycluster.eu/nsc-overview/nsc-structure/steering-group/.

5
https://nanopartikel.info/en/knowledge/operating-instructions/.

6
https://www.patrols-h2020.eu/publications/sops/index.php.
